# Control of Postharvest Green Mold in Citrus by the Antimicrobial Peptide BP15 and Its Lipopeptides

**DOI:** 10.3390/jof10120837

**Published:** 2024-12-03

**Authors:** Yu Lei, Aiyuan Lyu, Mengjuan Pan, Qingxia Shi, Haowan Xu, Dong Li, Mengsheng Deng

**Affiliations:** School of Biological Engineering, Sichuan University of Science & Engineering, Yibin 644000, China; yulei@suse.edu.cn (Y.L.); 322083202107@stu.suse.edu.cn (A.L.); 322095102429@stu.suse.edu.cn (M.P.); 323095102320@stu.suse.edu.cn (Q.S.); 323095102329@stu.suse.edu.cn (H.X.); dashtomlee@163.com (D.L.)

**Keywords:** peptide BP15, lipopeptides, green mold, mode of action

## Abstract

This study examined the efficacy and mechanisms of action of the antimicrobial peptide BP15 and its lipopeptides, HBP15 and LBP15, against *Penicillium digitatum*, the primary causative agent of green mold in citrus fruits. The findings revealed that all three antimicrobial peptides markedly inhibited the spore germination and mycelial growth of *P. digitatum*, with minimum inhibitory concentrations (MICs) of 3.12 μM for BP15, HBP15, and LBP15. The peptides induced morphological alterations in hyphae and elevated intracellular Sytox Green (SG) fluorescence signals, which is indicative of increased cell membrane permeability and disruption. This membrane damage was further supported by the heightened extracellular conductivity and the release of intracellular nucleic acid and protein. A gel retardation assay demonstrated that the peptides showed significant DNA binding and retardation effects. Furthermore, the peptides exhibited significantly lower hemolytic activity (*p* < 0.05) compared to commercial prochloraz in normal mammalian erythrocytes (sheep erythrocytes) at the tested concentrations. Therefore, BP15 and its lipopeptides, HBP15 and LBP15, show potential as effective agents for preventing green mold in citrus fruits.

## 1. Introduction

Citrus, a genus of the *rutaceae* family, is highly esteemed by consumers for its flavorful fruits and substantial nutritional value [1]. During postharvest storage, pathogen infections, particularly those caused by *Penicillium digitatum*, *Penicillium italicum*, *Geotrichum citri-aurantii*, and other pathogens, are the primary drivers of fruit decay [2,3]. Notably, green mold accounts for approximately 90% of postharvest citrus rot, resulting in serious economic losses for the citrus industry [4]. Chemical control remains the predominant strategy for managing postharvest infections in citrus fruit. However, this approach poses risks to human health and the environment, and it also fosters the development of pathogen resistance [5]. Therefore, there is an urgent need to identify novel and effective fungicides or alternative methods to control postharvest diseases in citrus fruits.

Antimicrobial peptides (AMPs) are small polypeptides naturally synthesized by organisms, serving as the primary defense against fungi, bacteria, and viruses [6]. Typically composed of 12 to 50 amino acid residues, usually with a net charge of 2–7 positive charge, AMPs exhibit multifunctional properties, including antimicrobial, antitumor, and wound-healing activities. Their applications span various sectors, such as food, agriculture, pharmaceutical industry, animal husbandry, and other fields [7,8]. The inhibitory mechanisms of AMPs are complex and not yet fully understood, but they primarily involve interactions between the cell membrane and intracellular targets [9]. At present, it is believed that the primary mechanism of AMPs is interaction with the target cell membrane, which increases the permeability of the membrane, destroys its integrity, and leads to the leakage of intracellular contents, thereby inhibiting cell growth and causing cell death [10]. The action on the membrane can be explained by models such as the barrel-stave, toroidal pore, carpet, and detergent-like models [11]. Wang et al. reported that the short cationic antimicrobial peptide PAF56 inhibits citrus pathogens by acting on the cell membranes and cell walls of fungi, resulting in their death [12]. At the minimum inhibitory concentrations, some AMPs do not significantly alter membrane permeability but still induce bacterial death, suggesting additional intracellular mechanisms. It has been proposed that AMPs can cross the cell membrane to interact with intracellular components, interfering with the normal cellular metabolism, including DNA transcription, protein synthesis, key metabolic enzymes, and cellular respiration [13]. For instance, the antimicrobial peptide zp37 binds to bacterial genomic DNA, inhibiting DNA replication and protein synthesis, thereby inhibiting bacterial growth [14].

There are two primary approaches for utilizing AMPs in disease management: one involves the chemical synthesis or genetic engineering of AMPs for direct application against pathogenic bacteria, while the other involves the genetic modification of biocontrol bacteria to express AMP-encoding genes, thereby eliminating pathogenic bacteria. Dou et al. demonstrated that the epsilon-poly-L-lysine (ε-PL) derived from *Streptomyces albulus* effectively controls blue mold in apples, with its antifungal mechanism elucidated though transcriptomic analysis [15]. CB-M derived from hemlymphatic of Mussel can inhibit the growth of *Botrytis cinerea* and reduce its incidence in tomatoes, cherry tomatoes, and grapes [16]. Ac-AMP2 and MiAMP1 derived from plants were successfully expressed in *Pichia pastoris* for biological control, with the recombinant yeast leading to the significant suppression of green mold in pears [17]. Although certain AMPs have been identified as effective in managing postharvest diseases, the limited number and inconsistent performance, so it is necessary to explore other AMPs in this domain.

BP15 (KKLFKKILKVL-NH_2_), a member of the CECMEL11 library, is a cationic short-chain polypeptide derived from the hybridization of the natural antimicrobial peptides cecropin A and melittin. Cecropin A is isolated from the hemolymph of the giant silk moth *Hyalophora cecropia* while melittin is separated and purified from honeybee (*Apis mellifera*) venom [18,19]. BP15 demonstrates efficacy against a range of post-harvest pathogens, including *P. digitatum*, *P. italicum*, *Stemphylium vesicarium*, and *Xanthomonas vesicatoria*, exhibiting notable antifungal activity in vitro [20,21]. The lipopeptides HBP15 (Hex-KKLFKKILKVL-NH_2_) and LBP15 (Lau-KKLFKKILKVL-NH_2_) are chemically synthesized peptides, and in the the N-terminal of BP15, they are covalently linked to hexanoic acid and lauric acid, respectively (Supplementary Figure S1). Given their amphiphilic properties, AMPs can strongly interact with negatively charged bacterial membranes. The incorporation of lipid tails of appropriate length at the N-terminals of AMPs, such as acetic acid, palmitic acid, and other fatty acids with inhibitory activity, enhances their hydrophobicity and potentially modulates their antimicrobial activity [22]. Among CGA-N9 the series of fatty acid-modified analogs, CGA-N9-C8C, which incorporates octanoic acid, exhibited the best inhibitory effects, stability, and biosafety against *Candida albicans* [23].

This study aimed to explore the inhibitory effects of BP15, HBP15, and LBP15 on *P. digitatum* and their potential mechanisms of action in order to broaden the application ranges of these peptides for postharvest green mold in citrus, and to provide new research ideas in the management of postharvest disease in citrus fruit.

## 2. Materials and Methods

### 2.1. Pathogens

The *P. digitatum* strain was sourced from the Laboratory of Microbial Agricultural Products Processing and Storage, Sichuan University of Science & Engineering (Supplementary Figure S2). The pathogen was cultured on potato dextrose agar (PDA) at 25 °C, and spores were collected after 7 days and diluted to form the required concentration spore suspension.

### 2.2. Synthesis of Peptides

All peptides were synthesized with over 95% purity by GenScript Corporation (Nanjing, China) and were stored at −20 °C. Additionally, the high-performance liquid chromatography (HPLC) and mass spectrometry (MS) images of the three peptides are shown in Supplementary Figure S3. BP15 and HBP15 were diluted with sterile water or dimethyl sulfoxide (DMSO), and LBP15 was diluted with DMSO. The maximum concentration of the primary batch was prepared using a solvent and then further diluted to the required concentrations.

### 2.3. MIC and MFC

The experiment was adapted from the method of Jia et al. [24]. A 180 μL spore suspension (1 × 10^4^ CFU/mL), containing 5% potato dextrose broth (PDB) by volume, was mixed with 20 μL of peptide solution in a sterile 96-well microplate and thoroughly mixed. The final peptide concentrations were 0.78, 1.56, 3.12, 6.25, 12.5, 25, 50, and 100 μM, with sterile water as the control. The mixtures were cultured in an incubator at 25 °C for 48 h, and the light absorption at OD_600nm_ was measured using an 800TS full-wavelength microplate reader (Bio Tek Instruments, Inc., Winooski, VT, USA). The minimum inhibitory concentration (MIC) was defined as the lowest peptide concentration completely inhibiting fungus growth for 48 h. Then, a 50 μL sample from the mycelium-free well was plated on PDA and left to culture at 25 °C for 48 h. The minimum fungicidal concentration (MFC) was the peptide concentration at which there was no pathogen growth after 48 h.

### 2.4. Mycelial Survival Assay

The mycelial survival rate was determined according to by Weerden et al. [25]. A spore suspension (5 × 10^4^ CFU/mL) was prepared with 5% PDB and incubated for 24 h at 25 °C. A mixture of 90 μL of spore suspension and 10 μL of peptide solution was added to in a sterile 96-well microplate; the final concentrations of the peptide were 0, 0.78, 1.56, 3.12, 6.25, 12.5, 25, 50 and 100 μM. The mixtures were cultured at 25 °C for 3 h before the addition of 10 μL of 3-(4,5-dimethylthiazol-2-yl)-2,5-diphenyltetrazolium bromide (MTT; 5 mg/mL; MACKLIN). The plates were then incubated in a dark environment for 16 h, followed by the addition of DMSO (100 μL). After mixing, the absorbance at 570 nm was subtracted from that of the background material at 690 nm, as measured using an 800TS full-wavelength microplate reader (Bio Tek Instruments, Inc., Winooski, VT, USA).

### 2.5. Scanning Electronic Microscopy (SEM)

The procedure was adapted from the method of Sun et al. with some adjustments [26]. The spore suspension and peptide solution were mixed to achieve final peptide concentrations of the MIC and 100 μM, respectively, and sterile water was used as a control. The mixture was incubated at 25 °C for 24 h. Mycelia were collected by centrifugation at 4000× *g* for 10 min, and the collected samples were prepared by adding glutaraldehyde fixative (pH 7.2–7.4, 2.5% *v*/*v*) at 4 °C overnight, followed by washing with PBS (pH 7.2) three times. Gradient dehydration was performed by sequentially adding 1 mL of 50%, 70%, 80%, and 95% ethanol for 10 min each. Samples were then treated with 100% ethanol twice and air-dried on an ultra-clean ventilated bench overnight. The samples were gold-coated and were observed using an SEM (Tescan VEGA 3SBU, Brno, Czech Republic).

### 2.6. Fluorescence Microscopy

The experiment protocol was adapted from Li et al. [27]. A spore suspension (10^5^ CFU/mL) of 90 μL was prepared with 5% PDB and incubated for 24 h at 25 °C. Subsequently, the peptide solution was added to final concentrations of the MIC, the MFC, and 100 μM, and the mixtures were left for 2 h, with sterile water as the control. After incubation, Sytox Green (Molecular Probes; KeyGen Biotech Co., Nanjing, China) was added at 4 μM and thte final concentration was 0.2 μM and was incubated for 5 min in the dark. The treated samples were washed with sterile water, resuspended in 20% glycerol solution, and prepared and observed under a DM3000 fluorescence microscope (Leica, Weztlar, Germany).

### 2.7. Measurement of Extracellular Conductivity

The experiment followed the method of Tao et al. with minor adjustments [28]. We assessed the impact of peptides on the fungal extracellular conductivity over time using a conductivity meter. A 100 μL spore suspension (1 × 10^5^ CFU/mL) was mixed with 10 mL of PDB into a centrifuge tube and cultured for 48 h at 25 °C and 160 rpm. After centrifugation at 4000× *g* for 15 min, the mycelium was washed, collected, and resuspended in 1.8 mL of sterile water. Peptides (200 μL) were added to achieve final concentrations of MIC, MFC, and 100 μM. The extracellular conductivity was measured at 0, 1, 2, 3, 6, and 9 h using a DDSJ-307F conductivity meter (INESA, Shanghai, China), with sterile water serving as the control.

### 2.8. Release of Intracellular Nucleic Acid and Protein

The experiments for measuring the intracellular nucleic acid and protein release were adapted from Paul et al. [29]. The impacts of antimicrobial peptides on intracellular nucleic acid and protein leakage in pathogenic fungi were determined using NanoDrop™. A mixture of 100 μL of spore suspension (1 × 10^5^ CFU/mL) and 10 mL of PDB were cultured for 48 h at 25 °C and 160 rpm. After centrifugation at 4000× *g* for 15 min, the mycelium was washed thrice with PBS buffer (pH 7.2), collected, and resuspended in 0.9 mL of PBS. Peptides (100 μL) were added to achieve the final concentrations of MIC, MFC, and 100 μM. At 0, 1, 2, 3, 6, and 9 h, the samples were centrifuged at 4000× *g* for 5 min, and 2 μL of centrifuged supernatant was used for NanoDrop™ (Thermo, Waltham, MA, USA) at OD_260nm_ and OD_280nm_ to measure intracellular nucleic acid leakage and protein leakage. Sterile water was used as a control.

### 2.9. DNA Binding Assay

In accordance with Miao et al. with slight adjustments, the gel retardation effect was analyzed [30]. The *P. digitatum* DNA was extracted according to the operation steps of the TIANGEN plant genome DNA extraction kit. The agarose gel electrophoresis utilized a 1% agarose concentration. The procedure involved adding 5 µL of peptide solution (3.12, 6.25, 12.5, 25, 50, and 100 μM) to 5 µL of DNA, respectively; adding 5 µL of sterile water to the control group and incubating the mixtures at room temperature for 30 min; and the subsequent addition of 2 µL of 6 × loading buffer to the peptide solution, followed by the and absorption of 5 µL of the loading sample. Electrophoresis was run at 110 V for 25 min, followed by the removal of the rubber plate and placement in the Tanon-2500B (Tanon Science and Technology Co., Ltd., Shanghai, China) for visualization.

### 2.10. Fruit Decay Test

This experiment followed the method of Zhou et al. with some adjustments [31]. The effects of three peptides on the control of green mold in citrus in vivo were studied by measuring the incidence rate and spot diameters on the citrus fruit. Mandarins were purchased from a local supermarket and selected for uniform size, ripeness, and a lack of mechanical damage. The mandarins were soaked in 75% alcohol for 30 s and then in 1% NaClO for 30 s, followed by rinsing with sterile water and drying with sterile paper to remove excess surface water. A sterile needle was used to drill holes near the equator (about 3 mm deep), and each mandarin was inoculated with 10 µL of 100 μM concentrations of three peptide solutions and 1 × 10^5^ CFU/mL *P. digitatum* suspension (*v*/*v* 1:1), compared with a mixture of equal parts sterile water and *P. digitatum* suspension. The mandarins were placed in a f container with sterile wet paper towels in order to preserve their freshness. A humid environment was maintained in the container, and the fruit was stored at room temperature. The incidence rate and spot diameter of the mandarins were measured in accordance with the method of Zhang et al. [32]. Each treatment group contained eight fruits, and each treatment was repeated three times.

### 2.11. Statistical Analysis

All the experiments were conducted in triplicates. Statistical analysis was conducted using Excel 2010, with data visualization in Origin 2021. The data were analyzed using SPSS 21.0, applying one-way ANOVA followed by Duncan’s multiple range tests, with significance set at *p* < 0.05.

## 3. Results

### 3.1. MIC and MFC

**Figure 1:**
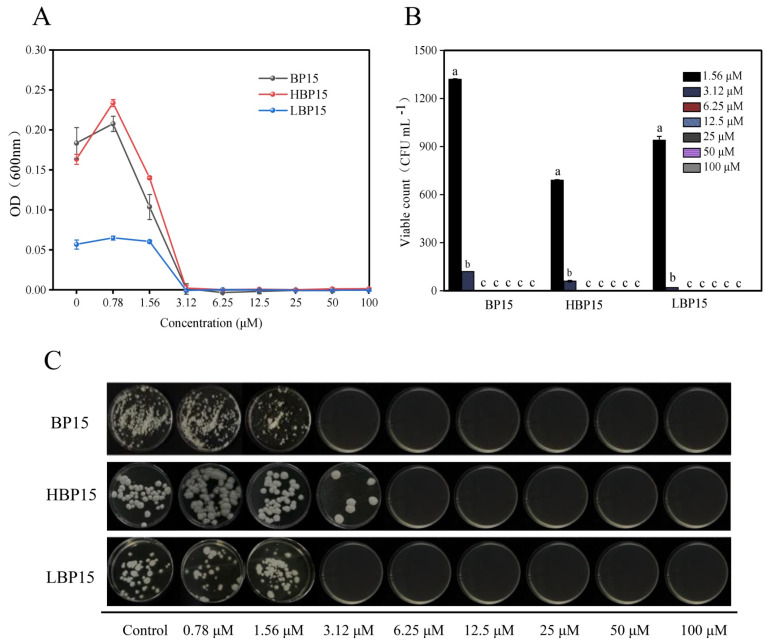
The antifungal activity of BP15, HBP15, and LBP15 against *P. digitatum* in vitro. (**A**) The data are shown as the means ± standard deviations of the OD_600nm_ values at different peptide concentrations after 48 h of incubation with *P. digitatum*. (**B**) Conidia of *P. digitatum* were incubated in PDA for 48 h at 25 °C alone (Control) or in the presence of peptides at 1.56, 3.12, 6.25, 12.5, 25, 50, or 100 μΜ. (**C**) Image shows viable colony counts in plates for each peptide concentration. Samples were repeated in triplicate. Bars labeled with the same letter are not significantly different according to Duncan’s multiple range test (*p* < 0.05).

As depicted in Figure 1, BP15 and HBP15 effectively inhibited *P. digitatum* growth at concentrations exceeding 0.78 μM, with the inhibitory effect intensifying as the concentration increased. No obvious inhibitory effect on *P. digitatum* growth was observed at LBP15 concentrations of 0.78 and 1.56 μM. The complete inhibition of *P. digitatum* growth was observed with BP15, HBP15, and LBP15 at concentrations of 3.12 μM, indicating that the MICs for *P. digitatum* were 3.12 μM for all three compounds.

Figure 1 further illustrates that increasing concentrations of BP15, HBP15, and LBP15 progressively diminished the survival of *P. digitatum* spores. The spore survival rate at 3.12 μM of BP15, HBP15, and LBP15 was significantly lower than that at 1.56 μM (*p* < 0.05). At concentrations of 6.25 μM, BP15, HBP15, and LBP15 eradicated *P. digitatum* spores, establishing the MFCs for *P. digitatum* as 6.25 μM for these compounds.

### 3.2. Mycelial Survival Rate

MTT is a widely utilized stain for the identification of cell viability. The mitochondrial enzymes in living cells can reduce exogenous MTT to blue-purple crystalline formazan, a property that renders MTT a valuable indicator of cell viability [33].

**Figure 2:**
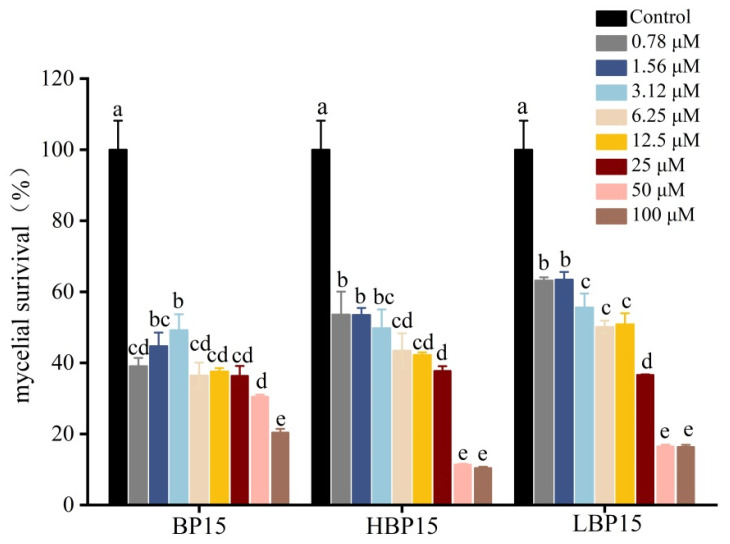
Effects of BP15, HBP15, and LBP15 on the activity of mycelia of *P. digitatum*. Data are expressed as the means of triplicate assays. Vertical bars represented the standard errors of means. Values followed by different letters were significantly different at *p* < 0.05.

Figure 2 demonstrates that the mycelial survival rate of BP15 at concentrations of 1.56 and 3.12 μM was higher than that observed at 0.78 μM. At a concentration of 100 μM, the mycelial survival rates for BP15, HBP15, and LBP15 were 20.38%, 16.31%, and 10.40%, respectively, significantly lower than those of the control (*p* < 0.05). Overall, the mycelial survival rate of *P. digitatum* decreased progressively with treatment duration across varying concentrations of BP15, HBP15, and LBP15, indicating that these peptides effectively inhibited *P. digitatum* mycelia.

### 3.3. Scanning Electron Microscopy (SEM)

**Figure 3:**
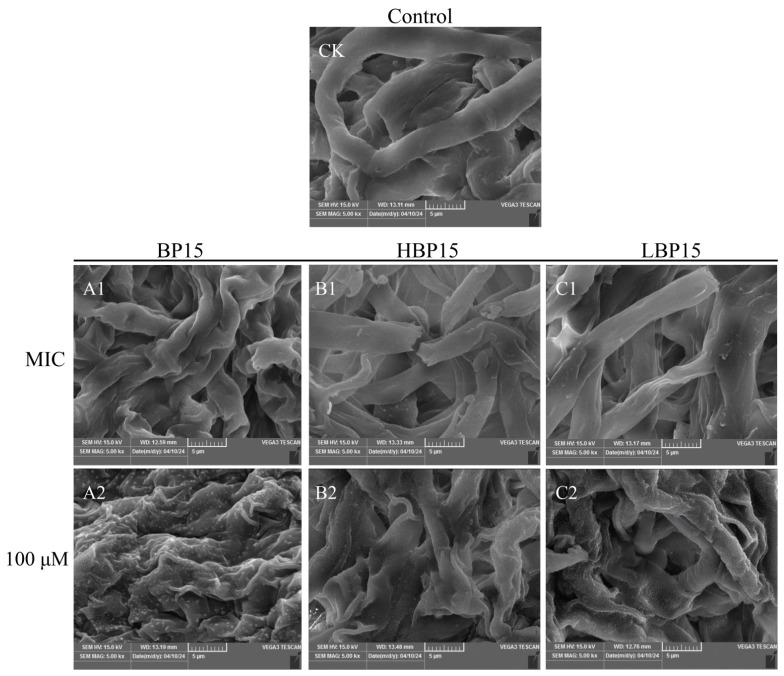
Effects of BP15, HBP15, and LBP15 on the mycelial morphology of *P. digitatum* observed by scanning electron microscopy. The *P. digitatum* mycelia were treated without peptides (CK) or with MIC (**1**) and 100 μM (**2**) of BP15 (**A**), HBP15 (**B**), and LBP15 (**C**), respectively. SEM HV: 15 kV; SEM MAG: 5 k×; bars = 5 μm.

Scanning electron microscopy (SEM) was employed to observe morphological alterations in mycelia treated with varying concentrations of BP15, HBP15, and LBP15. Figure 3 demonstrates that the control group (CK) exhibited a smooth mycelial surface and a normal mycelial shape, whereas peptide-treated mycelia exhibited varying degrees of damage. At a concentration of 3.12 μM, the mycelia of *P. digitatum* treated with the three peptides showed localized depressions, folds, and incomplete structures. Upon exposure to a high concentration of 100 μM, severe deformation and degeneration of the mycelia were observed, accompanied by the significant leakage of cell contents. Notably, mycelia treated with BP15 were almost entirely shrunken, and the extent of distortion and destruction was greater compared to that of those treated with HBP15 and LBP15.

### 3.4. Fluorescence Microscopy (FM)

Sytox Green (SG) is a nucleic acid dye that does not penetrate intact eukaryotic cells but enters cells with compromised membranes, binding to nucleic acids and emitting green fluorescence. This property makes SG valuable for assessing cell membrane integrity and the antibacterial efficacy of antimicrobial peptides [34,35].

**Figure 4:**
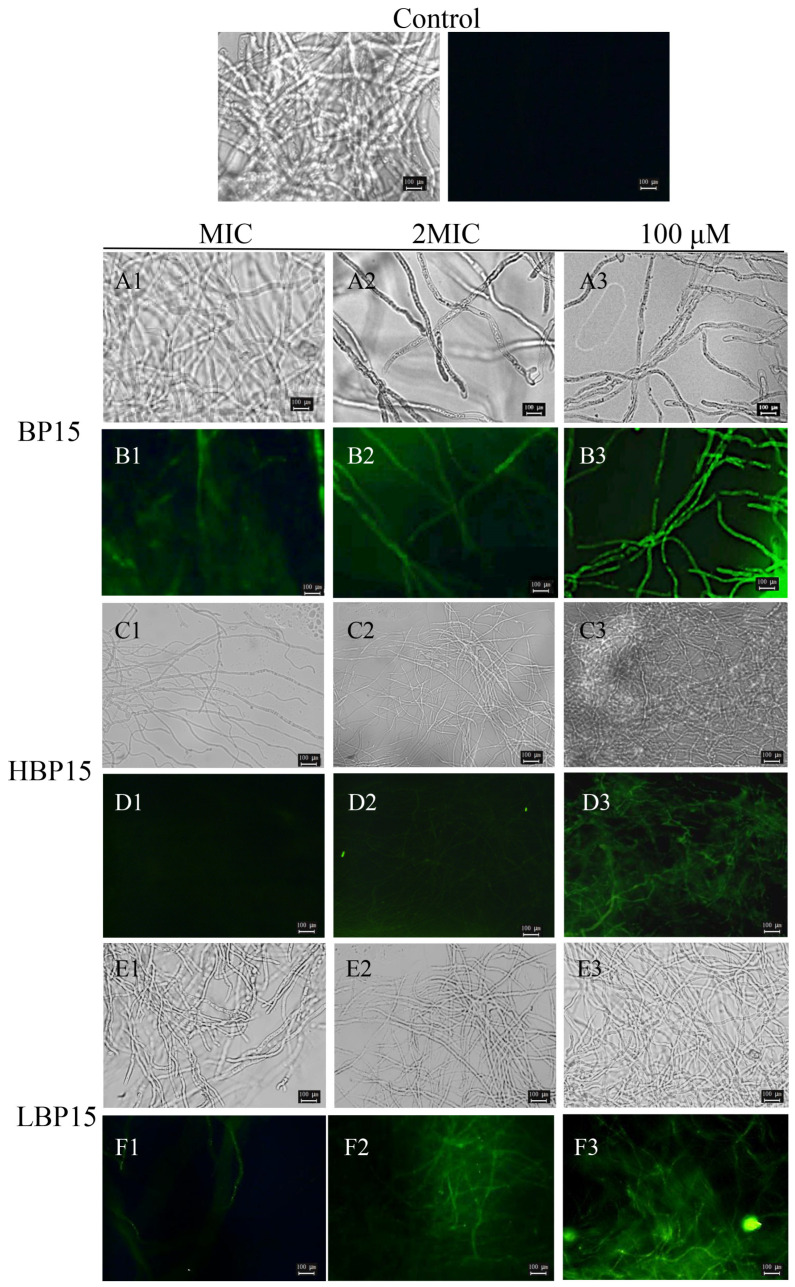
Effects of antimicrobial peptides BP15, HBP15, and LBP15 on the cell membrane permeability of *P. digitatum* mycelia. The *P. digitatum* mycelia were treated without peptides (CK) or with MIC (**1**), 2 MIC (**2**), and 100 μM (**3**) of BP15 (**A**,**B**), HBP15 (**C**,**D**), and LBP15 (**E**,**F**), respectively. CK1, (**A**,**C**,**E**) indicate bright light detection, and CK2, (**B**,**D**,**F**) indicate SG fluorescence detection. Samples were stained with SG and measured using a light fluorescence microscope. Magnification 40×; bars = 100 μm.

As depicted in Figure 4, no green fluorescence was observed in the control group (Figure 4 (CK)), indicating that the mycelial cell membrane of *P. digitatum* remained intact in the presence of sterile water. Upon treatment with the peptides at the MIC, weak green fluorescence was detected (Figure 4(B1,F1)), except in the HBP15-treated group (Figure 4(D1)), which showed no fluorescence. Increasing the peptide concentration to 2 MIC resulted in enhanced fluorescence intensity and continuity within the mycelia (Figure 4(D2,F2)), indicating increased membrane damage and reduced integrity. The BP15 treatment group exhibited a large but weak green fluorescence signal at 2 MIC (Figure 4(B2)). As the peptide concentration was increased to 100 μM, the BP15, HBP15, and LBP15 treatment groups displayed extensive continuous green fluorescence (Figure 4(B3,D3,F3)), indicating heightened membrane permeability. These findings demonstrate that the three peptides disrupted the integrity of the mycelial membrane.

### 3.5. Extracellular Conductivity

**Figure 5:**
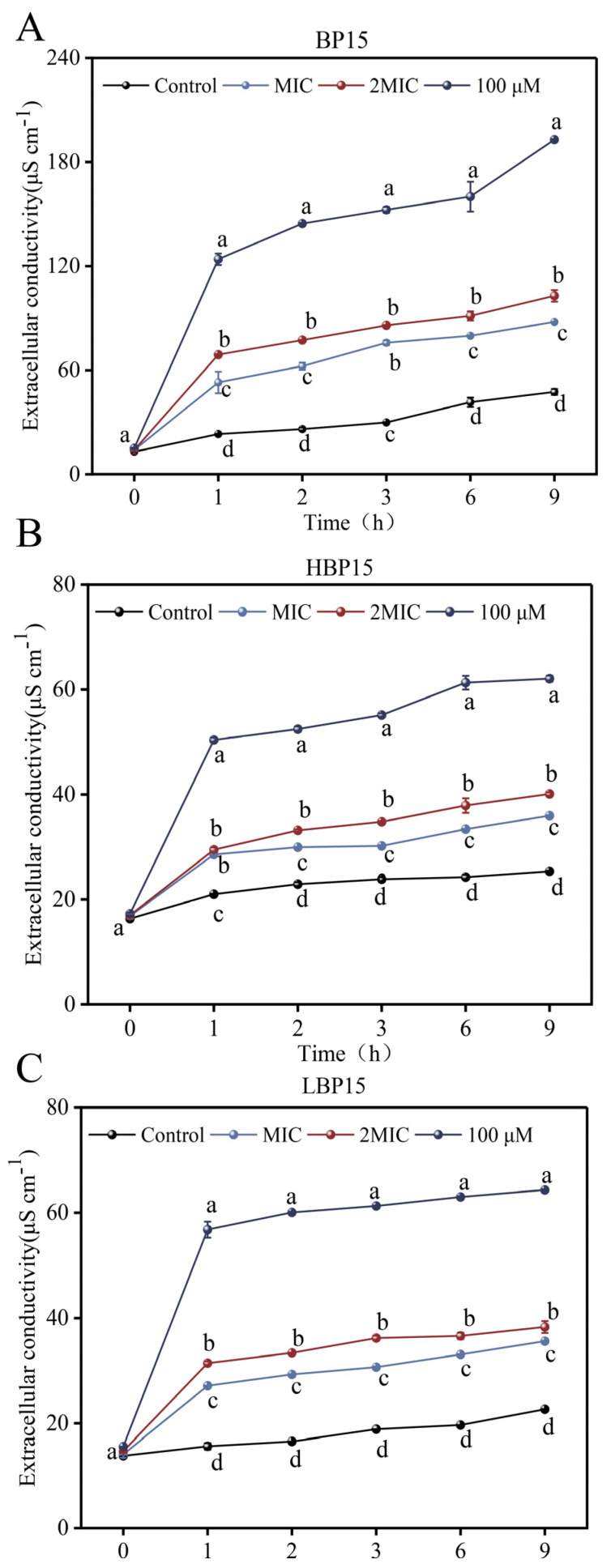
Effects of peptides BP15 (**A**), HBP15 (**B**), and LBP15 (**C**) on the extracellular conductivity of *P. digitatum.* Mycelia were mixed with BP15, HBP15, and LBP15 at MIC, 2 MIC, and 100 μM or in sterile distilled water (Control). Samples were prepared in triplicate, and the bars indicate standard deviations. Values followed by different letters are significantly different at the 0.05 level. The variance analysis was conducted using data from all the groups.

Figure 5 illustrates that the extracellular conductivity of *P. digitatum* exhibited a progressive increase with increasing treatment duration at varying concentrations of BP15, HBP15, and LBP15, peaking at 9 h post treatment. The conductivity of all the treatment groups increased rapidly within the first hour of culture, followed by a gradual rise thereafter. LBP15 did not result in a significant difference between the MIC and 2 MIC treatment groups at 1 h post-treatment, but both were significantly higher than the untreated group (*p* < 0.05). For the other treatment durations, all the groups showed significantly elevated conductivities compared to the untreated group (*p* < 0.05). Overall, all the peptides effectively inhibited *P. digitatum*, resulting in substantial changes in extracellular conductivity, with the effect on mycelial extracellular conductivity becoming more pronounced with an increase in peptide concentration. These results suggest that treatment with peptides may enhance the membrane permeability of *P. digitatum*, impairing its physiological function and thereby affecting mycelial growth.

### 3.6. Intracellular Nucleic Acid and Protein

**Figure 6:**
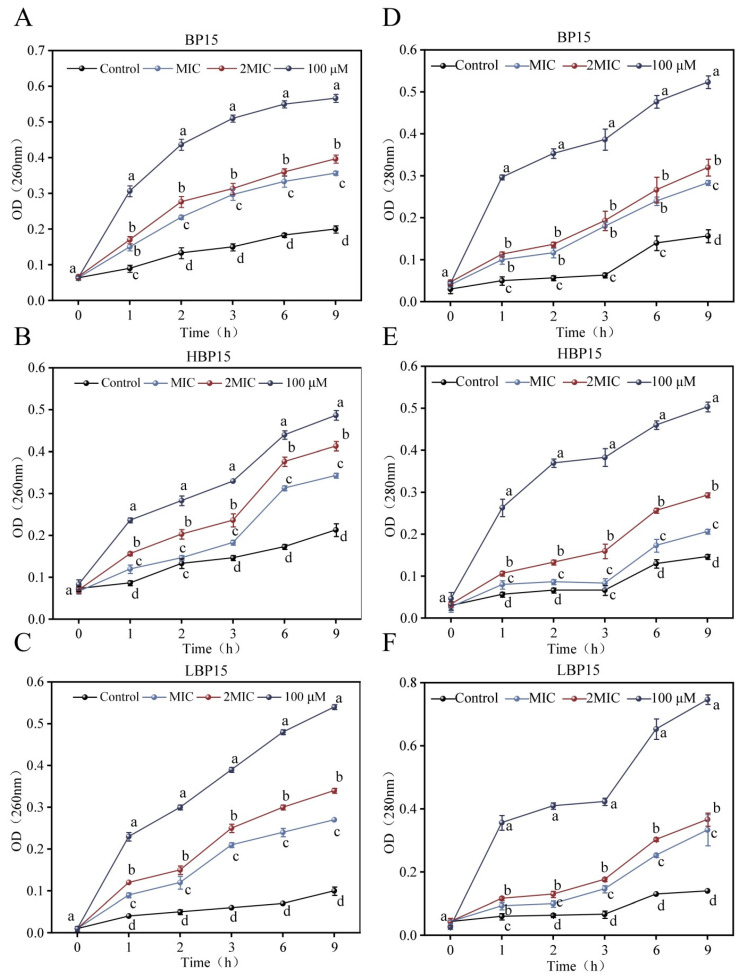
Effects of the peptides BP15, HBP15, and LBP15 on the release of intracellular nucleic acid (**A**–**C**) and protein (**D**–**F**) of in *P. digitatum.* Mycelia were mixed with BP15, HBP15, and LBP15 at MIC, 2 MIC, and 100 μM or in PBS (Control). Samples were prepared in triplicate, and the bars indicate standard deviations. Values followed by different letters are significantly different at the 0.05 level. The variance analysis was conducted using data from all the groups.

Figure 6 illustrates the intracellular nucleic acid leakage in *P. digitatum* following exposure to varying concentrations of the antimicrobial peptides BP15, HBP15, and LBP15. Higher OD_260nm_ values correspond to greater intracellular nucleic acid leakage, with each sample reaching a peak OD_260nm_ value 9 h post-treatment. At 1 h, BP15 treatments resulted in no significant difference between the MIC-treated and 2 MIC-treated groups (*p* > 0.05), but significant differences appeared at other time points (*p* < 0.05). At 2 h, HBP15 treatments resulted in no significant differences in OD_260nm_ between the MIC-treated and control groups (*p* > 0.05). Treatments with LBP15 resulted in significant differences in OD_260nm_ between the different treated and control groups (*p* < 0.05). Overall, all the peptides increased membrane permeability, leading to substantial changes in the intracellular nucleic acid level.

Additionally, Figure 6 depicts the intracellular protein leakage in *P. digitatum* mycelia treated with MIC, 2 MIC, and 100 μM peptides, as measured at 280 nm. Elevated OD_280nm_ values correspond to increased intracellular protein leakage, with each treatment group achieving peak OD_280nm_ values at 9 h post-treatment. At 1 and 6 h, BP15 treatments did not result in significant differences between the MIC and 2 MIC groups (*p* > 0.05). LBP15 result in no significant difference between the MIC and 2 MIC treatment groups at 1 h post-treatment (*p* > 0.05), but the protein leakage was notably higher than in the control group (*p* < 0.05). For the other treatment durations, all the groups showed significantly elevated protein leakages compared to the untreated group (*p* < 0.05).

### 3.7. DNA Binding

Gel retardation assays are routinely used to investigate the binding of AMPs to bacterial DNA. The formation of complexes between AMPs and DNA resulted in the reduced migration of DNA during electrophoresis or an increase in the number of complex molecules or even their retention at the origin, indicating that AMPs binding to pathogenic DNA [36].

**Figure 7:**
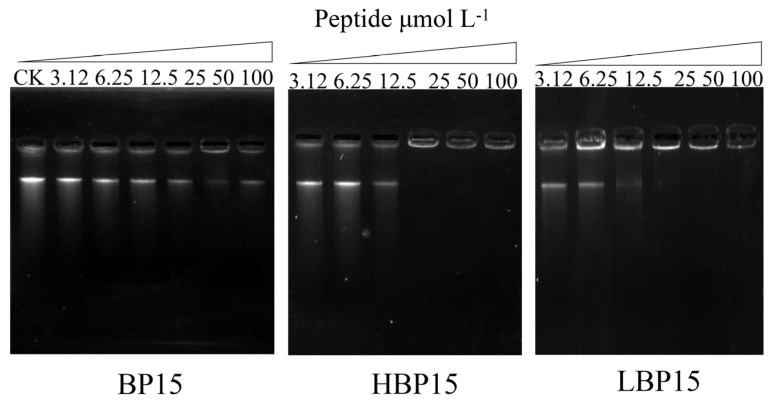
Gel retardation assay of the binding of peptides with *P. digitatum* genomic DNA. The *P. digitatum* DNA was mixed without peptides (CK) or with increasing amounts of peptides at 1:1 (*v*/*v*) for 1 h at room temperature and was analyzed via a gel retardation assay.

As illustrated in Figure 7, three peptides exhibited obvious blocking effects on the DNA of *P. digitatum* within the experimental range. At high peptide concentrations, competition with the nucleic acid dye for binding sites occurred, leading to the peptides binding to DNA instead of the dye. Specifically, when the concentrations of BP15, HBP15, and LBP15 were 100, 25, and 50 µM, respectively, the DNA bands were no longer visible, indicating the strong binding of peptides to DNA, resulting in the formation of large complex molecules that were retained at the origin. Consequently, HBP15 demonstrated a greater binding affinity for *P. digitatum* genomic DNA compared to LBP15 and BP15 within the experimental concentration range.

### 3.8. Fruit Decay Test

**Figure 8:**
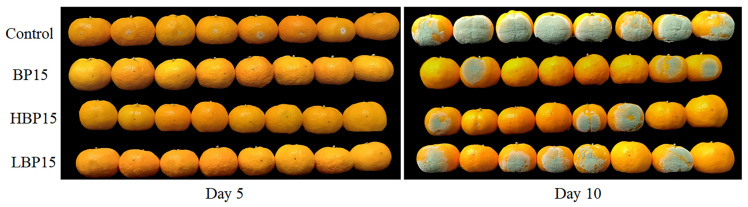
Effects of the peptides BP15, HBP15, and LBP15 on the infection of citrus by *P. digitatum*. Each mandarin was inoculated with 10 µL of 100μM concentrations of three peptide solutions and 1 × 10^5^ CFU/mL of a *P. digitatum* suspension (*v*/*v* 1:1), compared with a mixture of equal parts sterile water and *P. digitatum* suspension. Each column represents the mean of three replicates. Bars indicate the standard errors of the means. Values followed by different letters are significantly different at the 0.05 level. The variance analysis was conducted using the data from the same day. The photos show the symptoms of mandarins in each treatment group at 5 and 10 days after inoculation.

As shown in Figure 8 and Table 1, all three peptides controlled the incidence of green mold disease in mandarins to varying degrees. By day 5 of storage, the control group exhibited a 100% incidence rate. From the 5th to 9th day, the incidence and spot diameter in the BP15, HBP15, and LBP15 treatment groups gradually increased. Still, compared with the control group, all the peptides significantly reduced the incidence and spot diameter on mandarins (*p* < 0.05). By day 10 of storage, the spot diameter in the control group was 67.79 mm, with an extensive range of water stains and noticeable green mildew on the surface. The incidence of citrus in the LBP15 group was 87.50%, with no significant difference from the control group (*p* > 0.05). These results indicate that BP15 best inhibited green mold in citrus.

## 4. Discussion

With the abuse of antibiotics and the escalating resistance of pathogens, it is particularly urgent that we develop novel antimicrobial methods. Hence, it is particularly crucial to explore the mechanisms of antimicrobial peptides [10].

Conidia generated by *P. digitatum* are disseminated to the surface of fruits via wind, rain, soil dust, and other media. Upon landing on the surface or wound of the fruit, these conidia promptly germinate, leading to fruit decay. Hence, the lethal impact of antimicrobial peptides on spores is crucial for effective disease management [37]. Wang et al. demonstrated that the synthetic antimicrobial peptide PAF56 (GHRKKWFW) could inhibit the growth and sporulation of *P. digitatum* in postharvest green mold in citrus fruits, with a MIC of 8 μM for PAF56 against *P. digitatum* [12]. In the present study, the three peptides were found to effectively inhibit *P. digitatum* growth, kill spores, and reduce the survival rate of mycelia with MICs of 3.12 μM, thus outperforming PAF56. The enhanced efficacy may be attributed to the positively charged lysine in BP15, which has a greater tendency to adsorb into the cell membrane, causing the cell membrane to rupture or the formation of pores that expose internal cellular components, thus facilitating the entry of antimicrobial peptides and subsequent antifungal activity [38]. Additionally, the presence of leucine in BP15 enhances the affinity of antimicrobial peptides for phospholipid bilayers, aiding their embedding [39]. Previous research has shown that cationic antimicrobial peptides initially interact electrostatically with negatively charged bacterial membranes, and within a certain range, the appropriate net charge and hydrophobicity are positively correlated with the antimicrobial activity of AMPs [40].

As the primary defense mechanism of the cell, the cell membrane serves as a barrier, isolating the external environment from the internal structure while facilitating functions such as information transmission, substance exchange, and receptor binding [41]. It is currently generally believed that most AMPs primarily interact with cell membranes through various mechanisms, compromising membrane integrity, causing the leakage of intracellular small molecules, and thereby inhibiting the growth of pathogenic bacteria [8]. In this study, scanning electron microscopy (SEM) and fluorescence microscopy (FM) revealed that mycelia treated with BP15, HBP15, and LBP15 exhibited deformation and distortion, indicating enhanced membrane permeability and compromised integrity, with the extent of damage correlating with peptide concentration. When microbial cell membranes are compromised, their permeability significantly increases, leading to the leakage of crucial cellular components such as cytoplasm, nucleic acids, and ions [42]. This study measured the extracellular conductivity and intracellular nucleic acid and protein leakage in *P. digitatum* following treatment. The results demonstrated that peptides could destroy the cell membrane of *P. digitatum*, resulting in the loss of cell contents, the leakage of intracellular constituents, and a sharp increase in extracellular conductivity, thereby inhibiting cell growth. The 100 μM treatment resulted in the highest electrical conductivity and nucleic acid protein levels, similar to the effects observed with Thanatin, Ponericin W1, and Mastoparan-S on cell membranes [43].

AMPs have been reported to penetrate cells or undergo endocytosis, interacting with intracellular targets such as nucleic acids. This interaction can affect DNA or RNA synthesis and protein synthesis, disrupt the transfer of genetic information within the cell, and consequently interfere with normal bacterial metabolism [44,45]. MSI-1 not only increases membrane permeability but also directly interacts with DNA across the membrane, inhibiting DNA replication and protein expression, thereby affecting the normal metabolic activities of bacteria [46]. Gel retardation assays showed that BP15, HBP15, and LBP15 exhibit an obvious retardation effect on the DNA of *P. digitatum* within the experimental range, indicating that DNA binding is a complementary antifungal mechanism. This observation is consistent with the DNA binding effects reported for LRpG [47] and CB-M [16]. Nucleic acids are crucial for gene storage, replication, transcription, protein encoding, and maintaining cell integrity. It is hypothesized that the binding of the three peptides to DNA not only affects the growth of *P. digitatum* but may hinder physiological metabolism.

In vivo experiments showed that the three peptides could significantly inhibit the incidence and spot size of green mold in citrus fruits and had obvious control effects on the mold. This suggests that BP15, HBP15, and LBP15 can be expected to act as potential fungicides. The cytotoxicity of BP15, HBP15, and LBP15 was assessed via their hemolytic activity against sheep red blood cells. The findings indicated that the three peptides showed low hemolysis in the test range, and the hemolysis of normal mammalian red blood cells (sheep red blood cells) was significantly lower than that of commercial prochloraz (*p* < 0.05) (the results have not yet been published). However, when the mechanism of action of AMPs involves interaction with cell membranes, their cytotoxicity becomes a primary limitation for concurrent development. The low hemolytic activity of these three peptides may be indicative of an antifungal mechanism that extends beyond cell membrane disruption [48].

The results demonstrated that BP15, HBP15, and LBP15 exert multiple modes of action, with their inhibition of *P. digitatum* involving both cell membrane disruption and DNA interaction within the cells. The mechanism is analogous to that of TroHepc2-22 [49] and pBD2 [50]).

## 5. Conclusions

This study elucidated the mechanism by which the antimicrobial peptide BP15 and its lipopeptide derivatives, HBP15 and LBP15, inhibit *P. digitatum.* These peptides enhance cell membrane permeability, disrupt membrane integrity, and induce the leakage of nucleic acid and protein. Additionally, they bind to *P. digitatum* DNA, thereby impeding its normal growth. BP15, HBP15, and LBP15 exhibit potent efficacy in controlling green mold in citrus and show promise as novel broad-spectrum fungicides.

## Figures and Tables

**Table 1:** Effects of BP15, HBP15, and LBP15 on the infection of citrus by *P. digitatum*.

	Incidence Rate (%)				Spot Diameter (mm)			
	Control	BP15	HBP15	LBP15	Control	BP15	HBP15	LBP15
5 d	100.00 ± 0.00 ^a^	0.00 ± 0.00 ^d^	12.50 ± 0.00 ^c^	62.50 ± 14.43 ^d^	24.43 ± 3.21 ^a^	0.00 ± 0.00 ^c^	1.41 ± 0.80 ^c^	10.80 ± 4.93 ^b^
6 d	100.00 ± 0.00 ^a^	4.17 ± 7.22 ^c^	20.83 ± 7.22 ^c^	70.83 ± 19.09 ^b^	36.61 ± 2.88 ^a^	0.43 ± 0.74 ^c^	4.45 ± 0.80 ^c^	21.23 ± 4.46 ^b^
7 d	100.00 ± 0.00 ^a^	25.00 ± 12.50 ^c^	37.50 ± 0.00 ^c^	80.83 ± 11.54 ^b^	51.25 ± 2.88 ^a^	4.33 ± 2.49 ^c^	9.76 ± 0.84 ^c^	34.00 ± 10.39 ^b^
8 d	100.00 ± 0.00 ^a^	29.17 ± 7.22 ^c^	37.50 ± 0.00 ^c^	80.83 ± 11.54 ^b^	66.67 ± 3.55 ^a^	8.13 ± 4.24 ^c^	14.81 ± 1.19 ^c^	45.60 ± 13.19 ^b^
9 d	100.00 ± 0.00 ^a^	29.17 ± 7.22 ^c^	37.50 ± 0.00 ^c^	80.83 ± 11.54 ^b^	80.33 ± 3.21 ^a^	12.73 ± 5.65 ^c^	19.72 ± 1.95 ^c^	54.08 ± 12.44 ^b^
10 d	100.00 ± 0.00 ^a^	37.50 ± 12.50 ^b^	41.67 ± 7.22 ^b^	87.50 ± 0.00 ^a^	91.04 ± 4.59 ^a^	17.24 ± 9.03 ^c^	23.27 ± 3.61 ^c^	69.6 ± 8.37 ^b^

The results are presented as the means ± standard deviations. The letters ‘a’, ‘b’, ‘c’ and ‘d’ represent significant differences at the 0.05 level, which were analyzed using the data for the same pathogen on the same day.

## Data Availability

The original contributions presented in this study are included in the article/Supplementary Materials. Further inquiries can be directed to the corresponding author.

## Supplementary Materials

The following supporting information can be downloaded at: https://www.mdpi.com/article/10.3390/jof10120837/s1, Figure S1: The peptide BP15 (A), HBP15 (B), and LBP15 (C) structure; Figure S2: Phylogenetic tree based on ITS sequences; Figure S3: High-performance liquid chromatography (A–C) and mass spectrum (D–F) of the synthetic peptide BP15, HBP15, and LBP15.
