# Supplementary material for: Control of Postharvest Green Mold in Citrus by the Antimicrobial Peptide BP15 and Its Lipopeptides
Source: J Fungi (Basel). 2024 Dec 3;10(12):837. doi: 10.3390/jof10120837 (PMC11676762; doi:10.3390/jof10120837)
Supplement: Supplementary file 1 [file jof-10-00837-s001.zip › jof-3325638-supplementary.pdf]

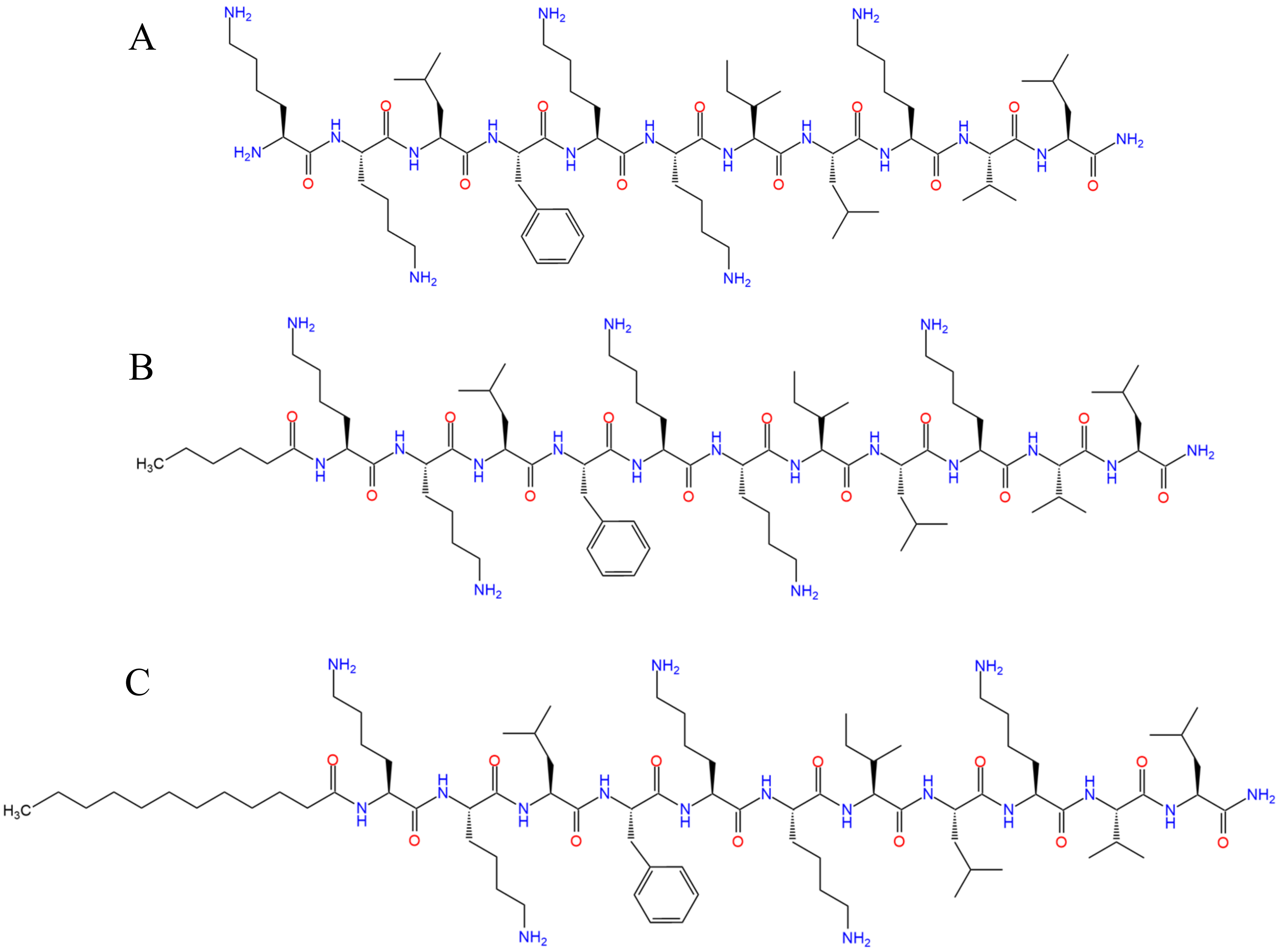

Figure S1. The peptide BP15(A), HBP15(B), and LBP15 (C) structure.

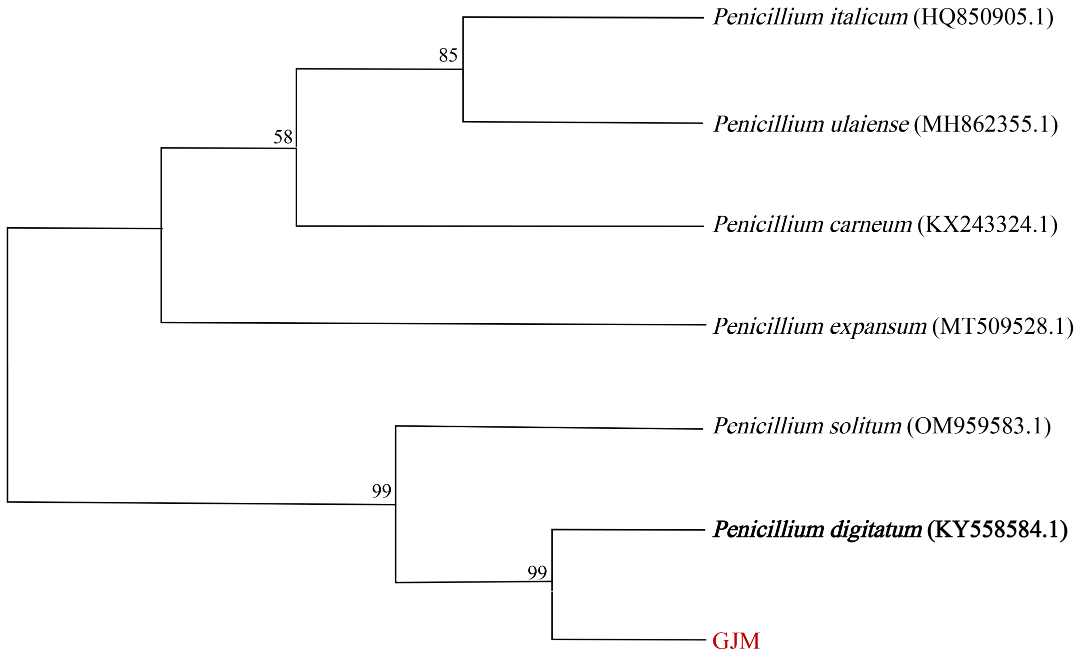

Figure S2. Phylogenetic tree based on ITS sequences.

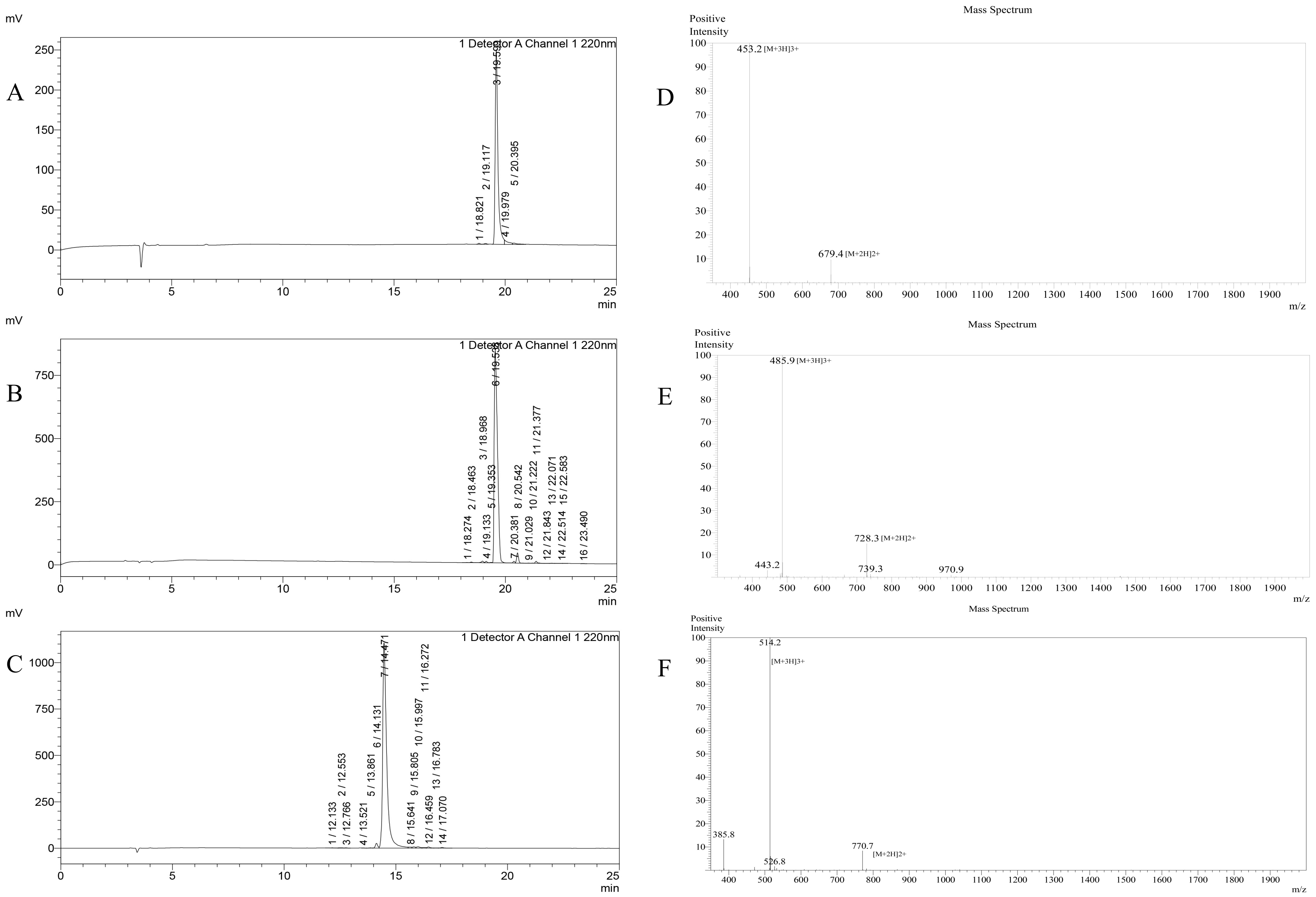

Figure S3. High-performance liquid chromatography(A, B, and C) and mass spectrum(D, E, and F) of the synthetic peptide BP15, HBP15, and LBP15.
